# Case series: Cystic degeneration in uterine leiomyomas

**DOI:** 10.4103/0971-3026.35820

**Published:** 2008-02

**Authors:** Chhavi Kaushik, Akhila Prasad, Yashvant Singh, BP Baruah

**Affiliations:** Department of Radiodiagnosis, Dr. Ram Manohar Lohia Hospital, New Delhi - 110 001, India

Uterine leiomyomas are the commonest gynecological neoplasms. The typical appearances of leiomyomas are easily recognized on imaging. However, the atypical appearances that follow degenerative changes may cause confusion in diagnosis. Here we present the USG and MRI findings in two different patients with uterine leiomyomas that had undergone cystic degenerative changes, mimicking a complex adnexal cyst of ovarian origin in one case and a large myometrial cyst in the other.

## Case 1

**Figure 1:**
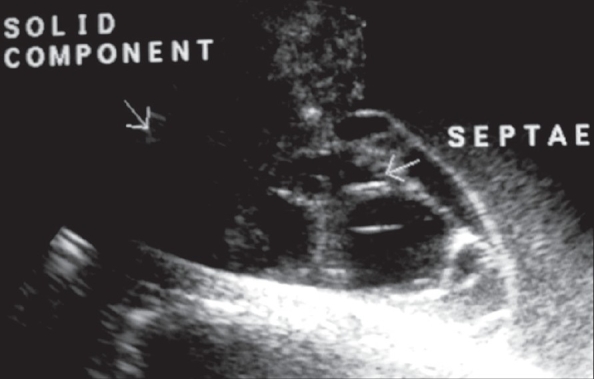
Transvaginal sonography showing a complex cystic mass with multiple internal septations in the right adnexal region

A 35-year-old woman presented with a history of lower abdominal pain and distension for a period of around 6 months. On abdominal examination, vague right abdominal fullness was felt. Per vaginal examination revealed an adnexal mass. A pelvic USG examination revealed a large, complex, predominantly cystic mass, approximately 10.0 × 8.5 × 7.0 cm in size, with multiple fine internal septae, arising from the right side of the pelvis and extending into the upper abdomen [Figure 1]. The uterine body appeared pushed towards the periphery and both ovaries could not be identified separately. The diagnosis of a complex adnexal cystic mass of probable ovarian origin was made.

**Figure 2:**
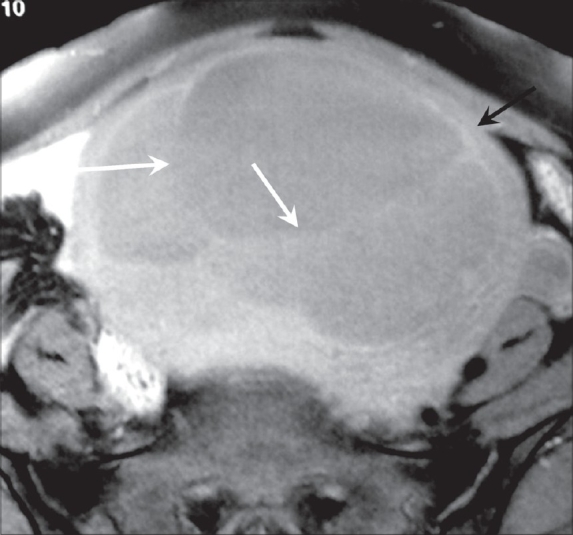
Fat-suppressed axial T1W MRI of the pelvis reveals a well-demarcated, thick-walled mass (black arrow), with the internal contents showing hypointense signal and multiple internal septae (white arrows)

**Figure 3:**
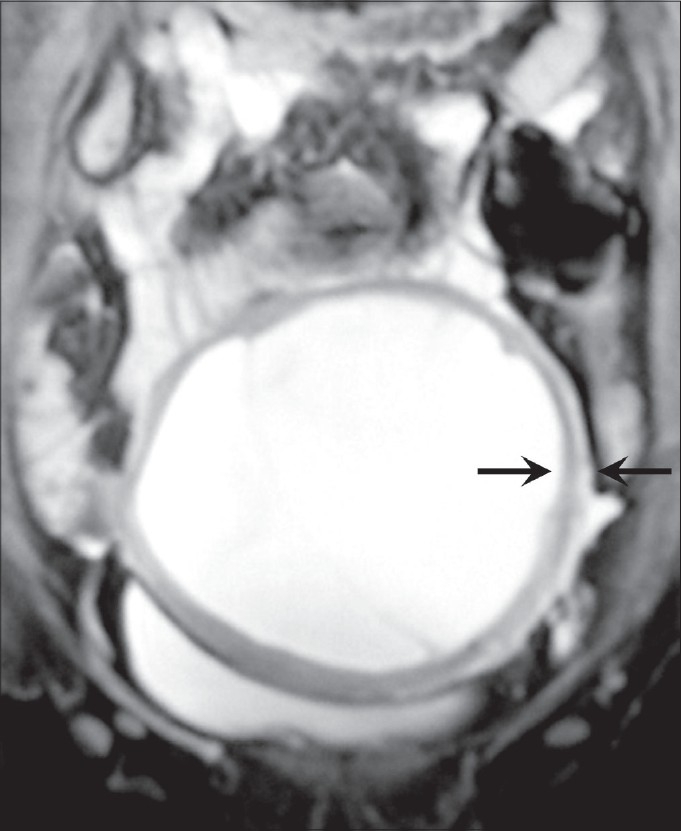
Coronal T2W fat-suppressed MRI shows a hypointense wall (black arrows) and hyperintense internal contents, suggestive of cystic degeneration

**Figure 4:**
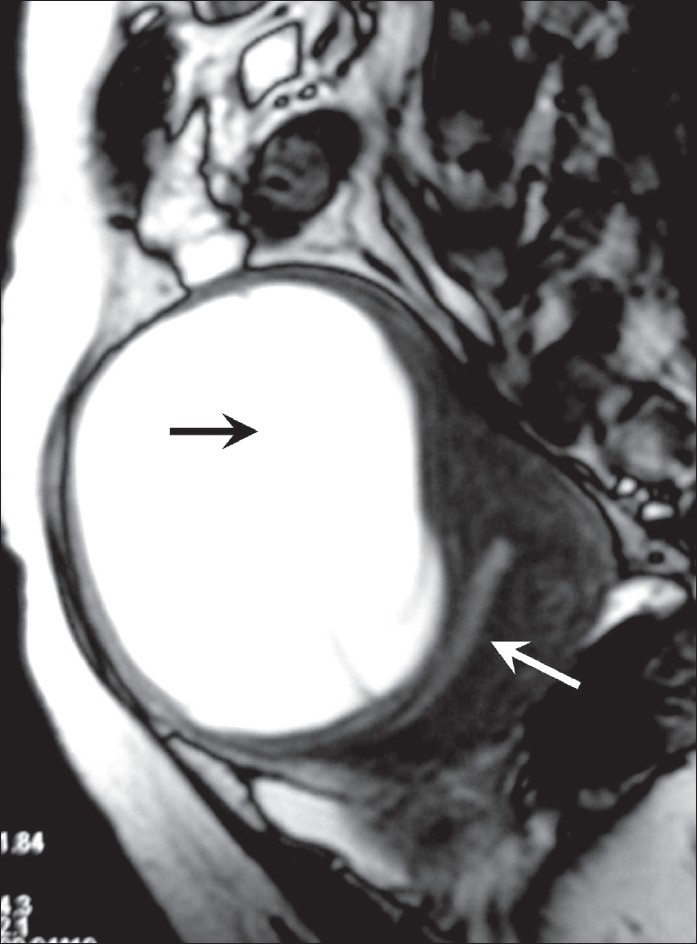
Gradient-echo MRI in the sagittal section shows a posteriorly displaced endometrial stripe (white arrow) and a cystic mass in the anterior myometrium (black arrow)

MRI showed a midline, well-circumscribed cystic mass, with multiple fine internal septae and signal intensities consistent with fluid. The images revealed continuity of the cyst wall with the remainder of the uterine myometrium, thus indicating a myometrial origin [Figures 2–4]. The signal intensity of the cyst wall followed the signal intensity of the uterine myometrium. The mass was seen to arise from the anterior uterine wall with slight posterior displacement of the uterine endometrium [Figure 4]. On the basis of these findings, subserosal myoma or an intra-ligamentary fibroid with cystic degeneration were considered as differential diagnoses.

The patient underwent laparotomy. A large mass was found to arise from the anterior uterine body; the ovaries were not involved. The mass was resected and this was followed by hysterectomy. Histopathological examination revealed a leiomyoma with extensive cystic degeneration.

## Case 2

**Figure 5:**
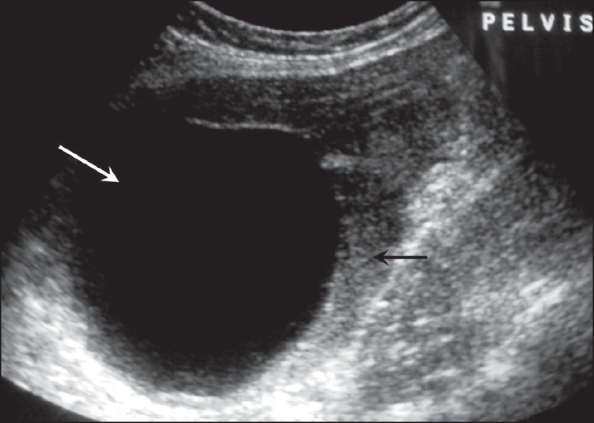
Transvaginal USG in the sagittal plane shows an intrauterine anechoic mass (white arrow) and the uterine myometrium (black arrow)

A 38-year-old lady presented with a history of long-standing menorrhagia. Abdominal and pelvic examinations were normal. USG evaluation revealed a well-circumscribed, anechoic lesion in the posterior uterine wall, measuring approximately 10.0 × 8.0 × 6.2 cm in size [Figure 5]. Both ovaries could be separately identified and were normal in size and echo pattern. The diagnosis of a uterine leiomyoma with cystic degeneration or a cystic adenomyoma of the uterus was made.

**Figure 6:**
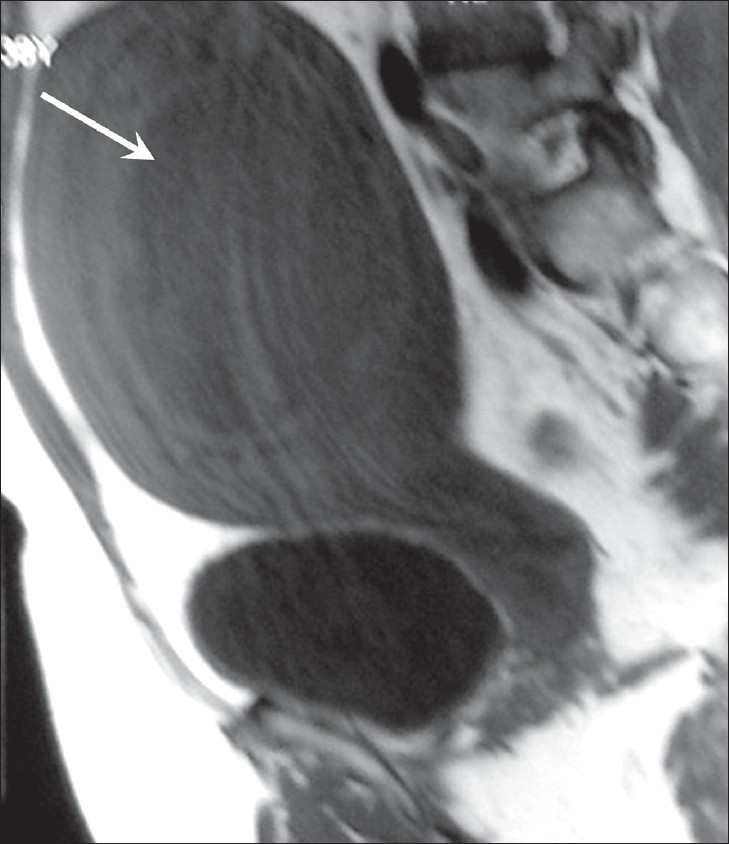
Sagittal T1W MRI reveals cystic degeneration in a hypointense intrauterine fibroid (white arrow)

**Figure 7:**
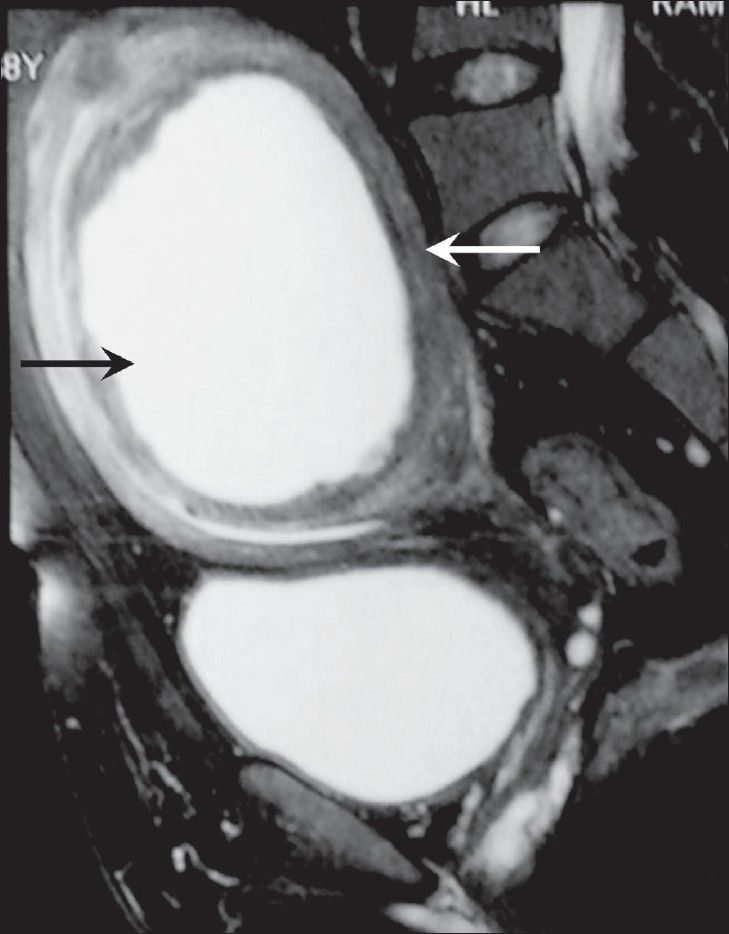
Coronal T2W fat-suppressed MRI shows a hypointense wall (white arrow) and hyperintense internal contents suggestive of cystic degeneration

MRI revealed a cyst with a hypointense signal on T1W images and hyperintense signal on T2W images [Figures 6 and 7]. The lesion showed an irregular outline and a few internal septae. Both ovaries could be identified and revealed normal signal intensities. The diagnosis of a uterine fibroid with cystic degeneration was made.

The patient underwent hysterectomy and histopathological examination revealed a uterine fibroid with marked cystic degeneration.

## Discussion

Leiomyomas are the commonest uterine neoplasms, occurring in around 20-30% of women in the reproductive age group.[1–3] They are composed of smooth muscle and fibrous tissue and are benign in nature.[1] Based on their location within the uterine wall, leiomyomas are classified into submucosal/subendometrial, intramural/myometrial or subserosal leiomyomas. The latter may be pedunculated and simulate adnexal masses.[1] It is a useful classification system as it relates to the clinical presentation and treatment options.[4] As leiomyomas enlarge, they may outgrow their blood supply, which results in various types of degeneration; these include hyaline, cystic, myxoid, or red degeneration and dystrophic calcification.[1,4] Hyalinization is the most common type of degeneration, occurring in 60% of tumors.[1] Cystic degeneration, observed in 4% of leiomyomas, may be considered an extreme sequel of edema.[1,2]

USG is the primary modality for diagnosing clinically suspected uterine fibroids.[4] USG commonly shows a hypoechoic or heterogeneous uterine mass, whose texture depends on the relative ratio of fibrous tissue to smooth muscle and the presence and type of degeneration.[1] Hence, Ieiomyomas may be minimally echogenic and irregular anechoic areas may be seen if cystic degeneration is present. Clusters of high-level echoes with distal acoustic shadowing are quite common with calcific degeneration.[1] Transvaginal USG provides better detail than transabdominal USG and can detect very small lesions and provide better differentiation of submucosal from mural lesions.[5] To differentiate between subserosal fibroids and adnexal masses, the ‘interface vessel sign’ may be of help. Seen both on color Doppler and MRI, tortuous vessels at the interface of the mass with the uterus indicate a uterine origin.[6] However, degenerative changes may result in heterogeneous or unusual presentations that may lead to a diagnostic dilemma.[4]

MRI plays a crucial role in determining the origin and nature of a pelvic mass in cases with inconclusive USG features.[5] MRI appearances of leiomyomas vary widely and may present a diagnostic problem.[7] On T2W images, leiomyomas are usually well-circumscribed masses which are sharply demarcated from the surrounding myometrium. Distinct low signal on T2W images is a typical MRI finding and is due to extensive hyalinization.[3,5] Degenerated leiomyomas show variable MR appearances. Red degeneration is characterized by the presence of a peripheral rim which shows low signal on T2W images and high signal on T1W images, corresponding to the obstructed veins at the periphery of the mass.[5] Cystic leiomyomas typically show decreased T1W and increased T2W signal intensities, with no enhancement of the cystic areas.[3] MRI is a useful imaging tool to demonstrate the pedicle or the presence of a normal uninvolved ovary - findings which are likely to enable a more accurate preoperative diagnosis.[1]

In conclusion, although fibroids usually have a characteristic USG appearance, degenerating fibroids can have variable patterns and pose diagnostic challenges. However, clinical and USG correlation, together with a knowledge of the variable USG appearances of degenerating fibroids, will generally lead to the correct diagnosis. A pedunculated, subserosal uterine leiomyoma with extensive cystic degeneration may mimic an ovarian tumor. MRI may be helpful in complicated cases.[2,3,7]
